# Exploring the Failed Implementation of an Entrustable Professional Activities-Based Curriculum for Pediatric Residency: “It’s What We Always Asked For, And Now Nobody Does It.” A Qualitative Study

**DOI:** 10.1177/23821205251370214

**Published:** 2025-09-04

**Authors:** Lena Moschinski-van Treel, Hans Martin Bosse, Lisa Marie Körner, Bernhard Steinweg, Janna-Lina Kerth

**Affiliations:** 1 Department of General Pediatrics, Pediatric Cardiology and Neonatology, Medical Faculty, 9170 University Children's Hospital Düsseldorf, Heinrich Heine University, Düsseldorf, Germany; 2 Medical Faculty, 9374 Rheinische Friedrich-Wilhelms-University, Bonn, Germany

**Keywords:** clinical competence, competency-based education, education, graduate, medical, internship and residency, medical, pediatrics

## Abstract

**Background:**

Medical education has been experiencing a transition from time- to competency-based. Since their introduction by Olle ten Cate in 2005, entrustable professional activities are a part of this process. We implemented a set of EPAs for the first 3 years of training at our hospital, encompassed by informational materials for trainees and supervisors.

**Objectives:**

Our objective was to assess barriers and facilitators for the implementation of entrustable professional activities in pediatric residency in a German tertiary hospital. Furthermore, our aim was to explore how they foster self-regulated learning and feedback, and to identify barriers and facilitators on the path to more confidence and safety.

**Methods:**

We applied a qualitative approach with focus-group discussions (adhering to COREQ guidelines), 3 each with residents and supervisors. Focus groups were recorded, transcribed verbatim, and de-identified. Data analysis was conducted based on qualitative content analysis.

**Results:**

Three months after the implementation, no resident had fulfilled the required assessments. In the discussions, we identified barriers to and facilitators of the process. Both may be attributed to internal and external factors. The concept was supported by residents and supervisors, but time, priorities in the clinical routine, lack of initiative, and an inhibition on the part of the residents to approach the supervisors were the main reasons for the concept to fail. We identified a higher degree of structure, as well as commitment and accountability, as possible facilitators.

**Conclusion:**

Entrustable professional activities are a promising means of promoting self-regulated learning and a positive feedback culture to increase confidence and safety in clinical practice. Our findings expose the factors that disrupt or promote this process. Our data provide guidance for implementation in a setting with little experience with this tool.

## Introduction

Medical education is experiencing a transition from time- to competency-based (CBME).^1^ Since their introduction by ten Cate in 2005,^2^ entrustable professional activities (EPAs) have become an important part of CBME both in under- and postgraduate settings.^3,4^ EPAs are units of professional practice describing the knowledge and skills necessary to perform a certain medical activity.^2^ They are “defined as tasks or responsibilities to be entrusted to a trainee once sufficient specific competence is reached to allow for unsupervised practice.”^5^ Thus, EPAs provide a framework to make specific judgements of trainee’s clinical competencies and to objectify entrustment decisions.^6^ This is an important point at all stages of medical education, especially to ensure patients’ safety.^7^ EPAs are also able to bridge the gap between education and patient-centered clinical routine,^8^ as well as gaps between different stages of education.^9,10^ Therefore, the Association of American Colleges (AAMC) introduced 13 Core EPAs for Entering Residency, defining tasks new residents should be able to perform without direct supervision.^11^

Pediatrics, with all its subspecialties, requires a diversity of clinical competencies from residents at early stages of postgraduate training. EPAs objectify this diversity of competencies and give residents a framework of activities they should be able to perform with or without supervision at certain stages. The American Board of Pediatrics has defined and published a body of 17 general pediatrics EPAs.^11,12^

In Germany, the Department of Pediatrics at the University Hospital of Bonn has implemented EPAs during the onboarding process of early residents^13^ and a framework has been developed for training in outpatient settings.^14^ In contrast to other countries, for example, the United States, there is generally little experience with structured operationalized programs in postgraduate medical education in Germany. The federal training catalogue is neither clearly operationalized nor time-bound, and lacks compulsory continuous formative feedback.^15^ Nevertheless, there also is an effort to create the path to structured and competence-based training programs, which emphasizes the importance of accompanying research in order to achieve a successful and sustainable change.^16^

EPAs represent a promising concept in the context of CBME, which, to our knowledge, has not yet been sufficiently investigated regarding its application in pediatrics. Therefore, our aim was to evaluate the use of EPAs in pediatric residency regarding feasibility, acceptance, and utility.

Our analysis of the literature revealed numerous studies describing at length the development process of sets of EPAs, both in Pediatrics and other fields.^17^ However, there is a paucity of literature on the implementation process, acceptance, feasibility, and assessment.^17^ This might well be because many centers experience difficulties during the implementation of EPA-based curricula. Furthermore, most of the available literature stems from North America.^3^ Except for the Netherlands, there is very little research about EPA implementation in Europe.^17^ Since there are regional and cultural differences in the structure of medical education, we believe it is essential to investigate medical education in different countries. We aim to contribute by investigating the implementation of EPAs in a German setting.

Our aim was to explore the feasibility, acceptability, and utility of EPAs in our setting. We aimed to investigate if EPAs could foster self-regulated learning by residents and a positive feedback culture among residents and supervisors. We sought to identify barriers and facilitators for the process to contribute to the body of evidence to gain a more thorough understanding of what works and what does not. We hope this will help other steering groups in the process.

## Methods

### Study design

To investigate our research question, we used a qualitative approach, conducting focus group discussions.^18^ This method was chosen for the following reasons: First, the implementation of EPAs in a preexisting team is influenced by the group context and social norms, which can be explored better in group discussions as opposed to individual interviews. We assumed that the interaction between participants would allow them to build on each other's thoughts and ideas and would lead to deeper insights into shared experiences. Lastly, focus groups research may be carried out more quickly and more cheaply than other methods, gathering a wide range of views in a shorter time compared to multiple individual interviews.^19^ We involved residents and supervisors to ensure depiction of both perspectives. We used the “Consolidated Criteria for Reporting Qualitative Research (COREQ): a 32-item checklist for interviews and focus groups” during planning, conducting, and analyzing the focus group discussions (Supplemental Material 1).^20^

### Setting

The study was conducted at the Department of General Pediatrics, Pediatric Cardiology and Neonatology at the University Children's Hospital Düsseldorf, Germany. The department comprises a total of 34 residents, 17 fellows, and 22 consultants. For each of the first 3 years of residency (one year each general in-patient care/Pediatric Emergency Room/Pediatric and Neonatal Intensive Care Unit), 15 EPAs specifically developed at our institution have been developed through a consensus procedure involving residents and supervisors (ie, consultants responsible for residents’ education during each rotation). These EPAs were implemented in September 2020 with the goal of completing a total of 12 per rotation. Each EPA included one to 3 practical skills that required direct supervision (ie, spinal tap, mask-valve-ventilation, and consultations of parents in specific settings) as well as cognitive learning objectives such as national and local guidelines.

### Participants

All residents and supervisors were invited via email and personally. We planned for at least 2 focus groups, consisting of 3 to 5 participants in each group. Focus group discussions were to be continued until saturation of data. Important characteristics of participants were collected using a short questionnaire.

#### Inclusion criteria

All residents in the first 3 years of training, that is, the period of training for which the EPAs were designed, and all fellows and consultants involved in the supervision of participants were eligible for participation.

#### Exclusion criteria

Participants who did not respond to the call for participation in the focus group discussions were not included. Residents in a period of training not included in the EPA program or on a research rotation were not eligible. Fellows and consultants without duties in the supervision of residents were not included

### Data collection

Focus groups discussions were conducted in November 2020 and took place in a hospital conference room. An interview guideline was developed to ensure structured moderation.^21^ The guideline covered the categories (self-regulated) learning, feedback, time, and structures during daily work, (self) assurance, and entrustment. The interview guideline was revised between discussions. Two members of the research team who are not involved in the supervision of EPAs moderated the groups. The 2 facilitators were female; one was a fourth-year resident (JLK), the other a medical student (LMvT). The resident was known to her peers and supervisors. Her involvement in the development and implementation process of the EPA curriculum was known to both residents and supervisors. Both had received training in conducting focus groups from another researcher who was well-experienced in the methodology (HMB) and were coached by this researcher throughout the process. The research team reflected regularly on how the interpersonal relations between the researchers and the participants in the study might have influenced outcomes. Despite the 2 interviewers and the participants, no other persons were present during the discussions. Both interviewers took notes during the focus groups. Pilot testing of the focus group discussion was not conducted due to the limited number of possible participants, but the interviewers met before and after each focus group to revise the interview guideline and to discuss which aspects should be explored further. All focus group discussions were video recorded, transcribed verbatim, and de-identified.

### Data analysis

Data analysis was conducted in an iterative process by the research team consisting of a resident (JLK), a supervisor (HMB), a medical student (LMvT) from our center, as well as a psychologist (LMK), and a medical education supervisor and pediatrician familiar with implementing EPAs (BS).

**Figure 1.**
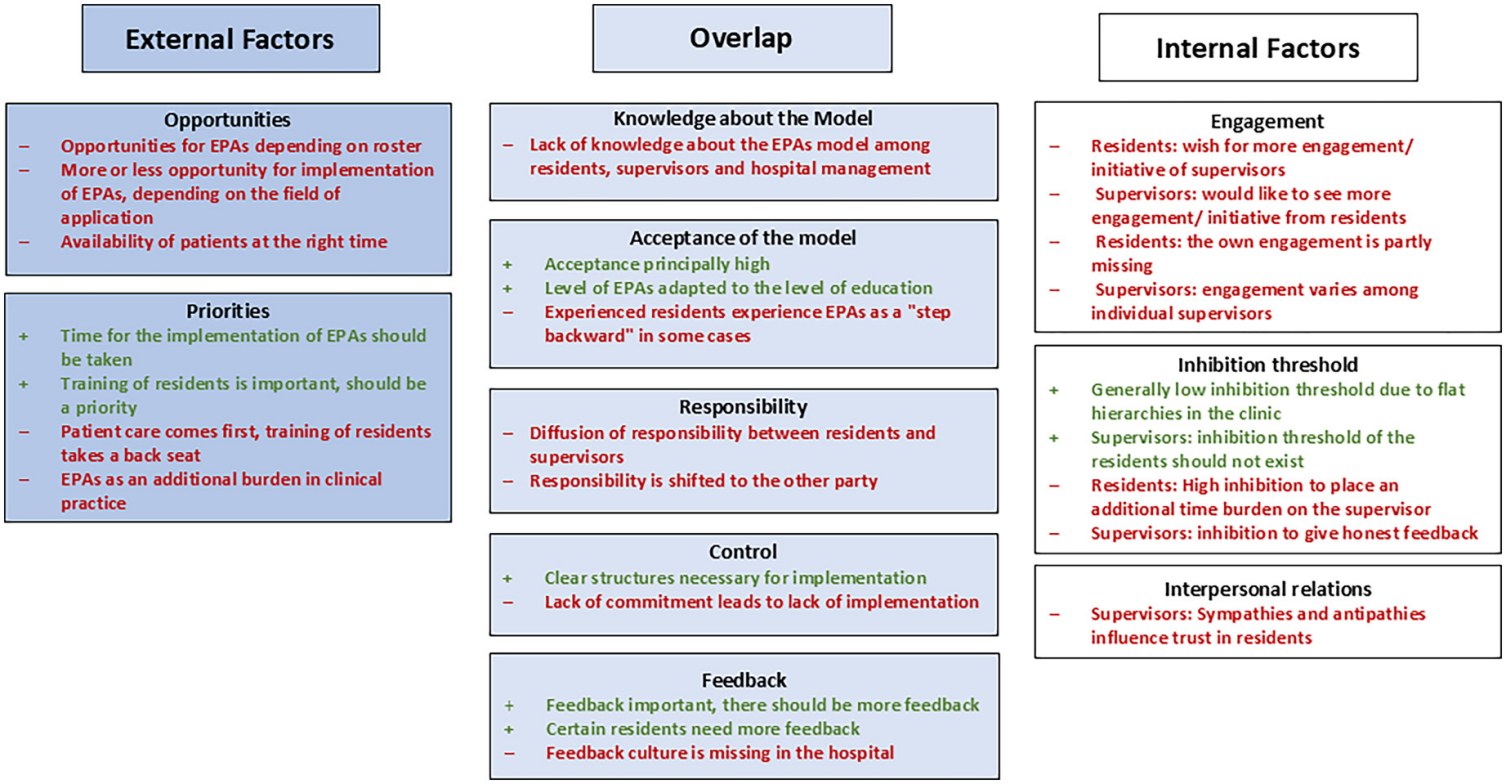
Barriers and facilitators influencing the implementation of entrustable professional activities (EPAs).

Data was coded by all researchers independently, and results were discussed within the research team. Themes were coded in an iterative process, all of them directly derived from data of the transcripts. Videos were additionally analyzed for the incongruency of content and facial expressions, and gestures. In summary, we aligned factors to an explicatory model (Figure 1).

Data saturation was monitored throughout the process. Data was coded immediately after each focus group discussion and discussed between the researchers to determine whether any new codes or themes had emerged. When no new themes surfaced during the discussions, it was discussed whether the questions needed to be rephrased. Data collection was stopped when saturation was reached.

Results were submitted to the participants for approval, and no significant new ideas were returned.

### Ethical approval and clinical trial registration

The study was approved by the Ethics Committee of the Medical Faculty of the Heinrich-Heine-University, Düsseldorf, Germany (2020-878) and was registered at the German register for clinical studies (DRKS00021094).

## Results

Our study aimed to investigate the feasibility, acceptance, and utility of EPAs in pediatric residency and their role on the way to enhance self-regulated learning and foster a competency-based medical education.

Residents and supervisors who agreed to participate were invited to attend a focus group discussion that suited their schedule. No potential participant refused to participate, but those who did not respond were not asked for reasons for nonparticipation. We conducted 6 focus group discussions, 3 with residents (*N* = 12 residents total) and 3 with supervisors (*N* = 7 supervisors total) until all researchers agreed theoretical saturation was reached.^22^ The focus group discussions lasted 34 min on average (26.5-45 min).

A total of 75% (*N* = 9) of the residents and 43% (*N* = 3) of the supervisors participating were female. The average duration of residency training at the time of the focus group discussions was 2 years (3-44 months). Residents had completed no EPAs, and supervisors had evaluated no residents; that is, nearly 3 months after the implementation of EPAs, no resident fulfilled the required 1 per month EPA-based assessments.

Through our focus group discussions, we determined barriers and facilitators influencing the implementation of EPAs (see Figure 1).

### External factors influencing the implementation of EPAs and self-regulated learning

#### Time resources

Time pressure is a well-known issue in the daily clinical routine of medical staff in acute patient care. Therefore, this also emerged as a core aspect that prevents residents from self-regulated learning and from performing EPAs as part of their daily routine.

Time pressure in the daily clinical routine was also named by supervisors as a determent.

F3T4, l.41ff: “I think one problem is the time management, especially in the emergency room I find it difficult to do this instead of everyday life, so to speak, because you never know when another ambulance will come, when the next patient will arrive.”

#### Structure and organization

Structure and organization of the daily work environment were barriers to the implementation of EPAs. In addition to a lack of dedicated time, treatment processes, general task management, and integration of education in daily clinical routines were often blamed for not having completed any EPAs.

#### Opportunities

The barriers regarding structure and organization included the lack of opportunity to complete EPAs, for example, due to a duty schedule with many night shifts or due to working on different wards repeatedly.

F2T3, l.235ff.: “So I think it's actually difficult to create that moment - there's a child who fulfills one of the criteria, the senior physician has time and the assistant has time. It seems really difficult for that to come together.”

F2T3, l.63ff.: “And especially when you are no longer on a fixed ward and rotate a lot and no longer have a senior physician as a contact person, it is of course a bit more difficult to know who to speak to.”

#### Priorities

In the daily routine, the focus lies primarily on providing the best possible care to patients and managing the ward well, which is why the residents tended to neglect structured and continued self-regulated learning and the completion of EPAs.

F5T3, l.16ff.: “The other reason why it hasn't worked so far is actually, I think, a pure personal omission, yes, that one is too comfortable, doesn't remember, that one has enough other things to do and so then these things, which somehow come on top of everything else, then simply fall behind, yes. And for that you then need even more, (pause) in a certain way pressure or obligation behind it, so that that is also really implemented, yes.”

Conversely, the mutual agreement between residents and supervisors that training should be prioritized, with time specifically set aside for it, represents a significant facilitating factor.

#### Internal factors

##### Residents’ initiative

Residents reported they need to show more initiative and responsibility so that the concept of EPAs can be implemented successfully and sustainably. One resident said, “in principle it is what we residents always, always ask for, […] now there it is and still nobody does it (laughs). So it's a little bit crazy.” [F2T3, l.70ff.]

F1T3, l.528ff.: “So I think, I think I'm also mad with myself that I haven't done that yet because I think that's really great and I think it's only going to be if we do that in the end and that's for us and for the future residents, but I think that's only going to work if we actually just do it that way.”

The supervisors also expected and asked for more initiative on the part of the residents.

F4T1, l.31ff.: “Yes, and of course we expect that there is simply also initiative, as [participant] said, that the residents approach us independently and that we don't run after them all the time.”

##### Engagement of supervisors

Residents also wished for more engagement from supervisors. They believed that the concept would be easier to implement if supervisors would also approach the residents occasionally and consider themselves responsible for the implementation of the EPAs to some extent as well.

F3T5, l.. 342f.: “I think it would be easier if they demanded it, so that they know you have to do it.”

##### Inhibition threshold

Some residents reported feeling inhibited about approaching supervisors for feedback or completion of an EPA. Even though the relationship with the supervisors was generally described as good, and, therefore, the inhibition threshold to approach them is generally low, residents were hesitant to place an additional burden on supervisors.

F2T2, l.131ff.: “I haven't done an EPA yet either, in retrospect it might have been possible at one point or another, but then it's kind of unfortunate to ask the senior physician at that moment if he's not free, because they're also often on the run.”

However, this point was negated by all supervisors. On the other hand, training of residents has a high value for supervisors, and they would like to see residents approach them more often. This openness towards residents might also be a facilitator.

F5T1, l.177ff.: “I also find it unusual that there. is the inhibition threshold to ask the senior physician, so I don’t think we are this stressed after all, On the other hand, I think it would be a pity if that, well, if that was really the case, that there were worries, because that is an important point, the training of our residents.”

##### Interpersonal relations

The trust that supervisors place in residents is currently strongly influenced by personal sympathy and experiences. There are residents whom supervisors generally trust more than others. EPAs could be a step in the direction of objectifying these trust decisions and making them less dependent on interpersonal relations.

F6T2, l.287ff.: “And then there are people who call me and I believe everything, so no matter on which topic they tell me something and there are people who call me, I believe nothing. So I have to be honest, I mean, what means I dońt believe anything, I would reconsider every assessment very carefully as to whether I agree with it or not, and others, if they tell me something, then I would say one hundred percent ‘okay, if that's how they see it, then that's how I see it, too’.”

F4T2, l.319ff.: “Yes, I think so. So there are a few points, a few things, a few emergencies that have to be treated somehow quickly, where I don't want to explain somehow at three in the morning what you should do, what he should do, but if you have talked it through beforehand with an EPA and he has perhaps also done it, that it then works in such a way that I say ‘do it, similar to what we did back then’ and then it is done and then it is easier and then you would also have more trust, yes of course. So I do believe that there are EPAs that can then strengthen the trust in an acute situation. Which then also not only give us the security or the confidence, but also the resident.”

### Factors including internal and external aspects

#### Knowledge about the concept

During the focus groups, it became clear that both residents and senior physicians were often uncertain about how EPAs exactly work, what the purpose behind the concept is, and how to use or integrate them in the clinical routine.

F1T1, l.156ff.: “Also that the senior physicians are well informed. For example, I've already spoken to two of them, one reaction was ‘please what?’, the other ‘yes, I've heard about it, I've been asked about it before, but I don't know what I have to do’. And that has of course also led to the fact that no EPA has taken place so far.”

#### Acceptance of the concept

It is also important that the concept of EPAs is generally accepted and supported by both the residents and supervisors. This aspect, which is an important facilitator, turned out to be fulfilled in our case. Both supervisors and residents shared a positive attitude towards the concept of EPAs and felt that the implementation in our center is possible, important, and a step in the right direction.

F2T3, l..45ff: “So I think that's a very, very good idea, especially in the first few months of residency, I think that's something I would have liked too.”

#### Responsibility/responsibility diffusion

Supervisors and residents tended to shift the responsibility for the implementation of the EPAs and education in general to each other—supervisors asked for initiative from residents while residents demanded more engagement and commitment from supervisors.

F4T1, l.155ff.: “Yes, I think that's always the case in these, that it's like that all the time, the residents want more training from us and we want the residents to show more initiative and to approach us actively, so to speak.”

Supervisors further noted that residency training used to function without structured frameworks like EPAs, questioning their necessity:

F6T2, l.41ff.: “even before any structures were introduced, the people who wanted to learn it, [they] learned it and the people who didn't want to learn it won't learn it properly even with the best structures.”

Although some supervisors recognized the potential benefit of implementing EPAs, they also expressed skepticism about whether such structures would influence their current trust in residents. Trust-based decisions about residents’ autonomy were described as largely informal and unstructured:

F6T2, l.287ff.: “… there are people who call me, and I trust them with everything, no matter on which topic; and there are people who call me and I don’t believe a thing.”

##### Commitment and control

Both residents and supervisors agreed that commitment and control regarding the EPAs are crucial for a successful implementation.

Although compulsion is generally avoided, residents as well as supervisors shared the opinion that some “pressure” is needed for the implementation of EPAs. Without any form of obligation, meaning a required, defined quantity of completed EPAs by the chief physician, for instance, it will be difficult to establish the implementation of EPAs.

F3T1, l.58: “I'm always a bit bothered by the compulsion, so you have to do it like this now, but I think otherwise it just doesn't work, so I can at least say about myself that it doesn't work then.”

F5T1, l.91f.: “So I think a certain pressure just has to be there for it to work in the end.”

Time set aside during the day specifically for EPAs was named as a possible facilitator by both groups.

##### Feedback

Residents reported that they get very little feedback in their training and that they generally would like to receive concrete, constructive, and critical feedback from their supervisors.

Furthermore, receiving feedback influences the residents’ self-regulated learning by providing an incentive to revisit certain content, which we identified as a strong facilitator and motivator for the implementation of EPAs.

The supervisors also confirmed that they could give more feedback, and the feedback culture in general should be improved. They noted that it is often difficult for them to give constructive and critical feedback. In this context, EPAs could be a good opportunity to provide more feedback and could be an aid in providing more objective and constructive feedback.

F2T2, l.88ff: “But I think it's good with the EPAs that you get feedback on concrete things or decisions that you would make in the situation, I think that's good. And also in general that this is provided for, because I think a feedback culture is important, also that you know what you can do better. I think, for example, that that comes too short in part in the training.”

F6T2, l.136ff.: “Yes, so there is little feedback, there is at least, there is at least also very little honest, constructive feedback since I can think, so. And actually, it's also difficult, I also find it difficult to give honest, constructive feedback, because, yes, because of course it's always a fine line between putting down and yes and just sort of constructive criticism.”

## Discussion

EPAs lead the way to competency-based continuing education in medicine. Although all involved in our EPA curriculum viewed this concept as promising and useful, the implementation (at least temporarily) failed in our setting. We seized the opportunity to identify significant potential barriers and facilitators for the implementation of EPAs on several different levels (see Figure 1), and we discuss their impact on an intended process of self-regulated learning with a lived feedback culture.

### Structure and control

In our focus group discussion, residents as well as supervisors called for more external control and a higher degree of structure in the process of EPA implementation. Continuing medical education in Germany hardly has any specified operationalized structure so far, a factor generally impeding the implementation of EPAs.^15^ Both residents and supervisors noted this lack of structure and expressed their desire for a more operationalized residency curriculum.

Schumacher et al^23^ identified specific facilitators and inhibitors to assessing EPAs in pediatric residency. The facilitators and inhibitors are assigned to 4 thematic areas, 2 of which were also addressed in our focus groups; firstly, the exposure of gaps in residency training through EPA implementation and, secondly, the engagement of faculty, which was also seen as an important factor in our study.

Schumacher et al also showed that Clinical Competency Committees (CCCs) have a strong influence, and while in some cases they may provide barriers, their favorable impact seems unquestioned. While there are no CCCs in our setting, both residents and supervisors expressed a strong wish for a sound structural basis for the EPA-based curriculum and assessment, which could be provided by such committees. As there are few operationalized regulations in the German residency programs, an integration of this concept would be challenging and would need to be tested in our setting.

Carraccio et al^24^ identified 10 core components of EPA implementation as a guide and an evaluation tool for local EPA-based programs. The authors stress that implementing EPAs is a highly complex undertaking in which, again, CCCs seem to play a crucial role as they are named in 5 out of the 10. This study assumes a preexisting supportive structure for the implementation of EPAs, which is missing in settings with little experience in competency-based medical education programs.

There is a paucity of data relating to the implementation and assessment of EPAs, and studies are most frequently conducted in the United States and Canada.^3^ In summary, findings in “highly developed countries” regarding competency-based medical education as the United States, Canada, and Australia, may not apply to countries where EPA programs have been less frequently implemented, such as Germany.

### Initiative/responsibility diffusion

The tension between supervisors and residents regarding initiative and engagement reflects deeper systemic and cultural dynamics in medical training. Supervisors expect more initiative from the residents. On the other hand, all residents mentioned they would appreciate more engagement from the supervisors—suggesting a mutual attribution of blame for the failure of EPA implementation. This reciprocal expectation may reflect a learned behavioral pattern passed on from one generation to the next. The supervisors’ reluctance to embrace EPAs may be rooted in their own training experiences, which lacked formalized structures yet led to high levels of competence. This history contributes to skepticism about the added value of EPAs and reinforces reliance on subjective, experience-based trust.

The unspoken implication that structural change questions supervisors’ past success may contribute to resistance. Entrustment decisions are already being made, but in an unstructured and implicit manner. While EPAs offer a more formalized approach, some supervisors doubt whether this would alter their deeply ingrained, intuitive methods of assessing readiness for responsibility.

As Peters et al suggest, supervisors often hesitate to grant learners greater autonomy, even when objective data support it.^25^ However, they also argue that entrustment decisions are inherently subjective and based on accumulated experience. In this light, the purpose of EPAs is not to eliminate subjectivity but to provide a broader and more consistent understanding of a resident's skills and entrustability.^26^

### Limitations

We carefully considered potential confounding factors throughout the study. We intended to avoid a potential social desirability bias regarding participants’ contributions and, therefore, did not provide feedback from residents’ interviews to senior staff or colleagues. We, therefore, did not include demographic information other than gender and duration of training in order to ensure that participants cannot be identified in a comparatively small cohort. A potential confounder may be the professional relationship between one of the 2 members of the research team who led the focus group discussions and the participants. The participants know 2 researchers personally. We actively encouraged participants to speak freely. There is no dependence between the participants and the members of the research team, particularly no influence on their career progress. Both facilitators were women, potentially inducing a gender bias. We specifically and reiteratively reflected on such issues from multiple perspectives. The number of participants was limited, but a saturation was shown.

Our study was conducted in a single institution and may not be generalizable, but—due to its temporary failure—could serve as an exemplary case.

## Conclusion

EPAs lead the way to competency-based continuing education in medicine. In our study, the participants generally saw this concept as promising and useful. However, the implementation of EPAs failed in our setting. We highlight potential barriers and facilitators for the implementation of EPAs. In identifying these barriers, our findings may support decision makers in other settings with comparatively little supportive infrastructure for and experience with EPAs. This will facilitate the intended process of self-regulated learning through the implementation of EPAs, fostering an emerging lived feedback culture. Thus, we contribute to shaping the future of continuing medical education to ensure that healthcare professionals are equipped with the knowledge and skills to deliver high-quality medical care.

## Supplemental Material

sj-docx-1-mde-10.1177_23821205251370214 - Supplemental material for Exploring the Failed Implementation of an Entrustable Professional Activities-Based Curriculum for Pediatric Residency: “It’s What We Always Asked For, And Now Nobody Does It.” A Qualitative Study Supplemental material, sj-docx-1-mde-10.1177_23821205251370214 for Exploring the Failed Implementation of an Entrustable Professional Activities-Based Curriculum for Pediatric Residency: “It’s What We Always Asked For, And Now Nobody Does It.” A Qualitative Study by Lena Moschinski-van Treel, Hans Martin Bosse, Lisa Marie Körner, Bernhard Steinweg and Janna-Lina Kerth in Journal of Medical Education and Curricular Development
