# Supplementary material for: Exploring the Failed Implementation of an Entrustable Professional Activities-Based Curriculum for Pediatric Residency: “It’s What We Always Asked For, And Now Nobody Does It.” A Qualitative Study
Source: J Med Educ Curric Dev. 2025 Sep 4;12:23821205251370214. doi: 10.1177/23821205251370214 (PMC12411700; doi:10.1177/23821205251370214)
Supplement: sj-docx-1-mde-10.1177_23821205251370214 - Supplemental material for Exploring the Failed Implementation of an Entrustable Professional Activities-Based Curriculum for Pediatric Residency: “It’s What We Always Asked For, And Now Nobody Does It.” A Qualitative Study [file sj-docx-1-mde-10.1177_23821205251370214.docx]

| **Domain 1: Research team and reflexivity** | | |
| --- | --- | --- |
| **Personal Characteristics** | | |
| 1. Interviewer/facilitator | Which author/s conducted the interview or focus group? | LMvT and JLK |
| 2. Credentials | What were the researcher’s credentials? E.g. PhD, MD | LMvT: medical student HMB, BS, JLK: MD LMK: PhD |
| 3. Occupation | What was their occupation at the time of the study? | LMvT: medical student JLK: resident HMB: consultant pediatrics BS: medical education administrator, consultant pediatrics LMK: pediatric clinical psychologist |
| 4. Gender | Was the researcher male or female? | LMvT, JLK, LMK: female HMB, BS: male |
| 5. Experience and training | What experience or training did the researcher have? | HMB is highly experienced in qualitative studies in the field of medical education, both in conducting the interviews and analyzing and interpreting the findings JLK had some prior experience; both LMvT and JLK received extensive training prior to and coaching during the process. BS and LMK have had some prior experience with data analysis |
| **Relationship with participants** | | |
| 6. Relationship established | Was a relationship established prior to study commencement? | Yes, most of the research team was known to participants |
| 7. Participant knowledge of the interviewer | What did the participants know about the researcher? e.g. personal goals, reasons for doing the research | HMB and JLK had been active in the process of developing and implementing EPAs and were known to participants in this role |
| 8. Interviewer characteristics | What characteristics were reported about the interviewer/facilitator? e.g. Bias, assumptions, reasons and interests in the research topic | Data analysis was conducted in an iterative process by the research team consisting of a resident (JLK), a supervisor (HMB), a medical student (LMvT) from our center as well as a psychologist (LMK), and a medical education supervisor and pediatrician familiar with implementing EPAs (BS). |
| **Domain 2: study design** | | |
| **Theoretical framework** | | |
| 9. Methodological orientation and Theory | What methodological orientation was stated to underpin the study? | Content analysis |
| **Theory** | | |
| **Participant selection** | | |
| 10. Sampling | How were participants selected? e.g. purposive, convenience, consecutive, snowball | All possible participants were approached |
| 11. Method of approach | How were participants approached? e.g. face-to-face, telephone, mail, email | Face-to-face and email, possible participants were asked to choose a date/time for participation in a focus group discussion. Some reminders were sent out. |
| 12. Sample size | How many participants were in the study? | n=12 residents and n=7 supervisors |
| 13. Non-participation | How many people refused to participate or dropped out? Reasons? | No active refusal; not all possible participants responded. Researchers did not ask for reasons for non-participation. |
| **Setting** | | |
| 14. Setting of data collection | Where was the data collected? e.g. home, clinic, workplace | workplace |
| 15. Presence of non-participants | Was anyone else present besides the participants and researchers? | no |
| 16. Description of sample | What are the important characteristics of the sample? e.g. demographic data, date | 75% (n=9) of the residents and 43% (n=3) of supervisors participating were female. Average duration of residency training at the time of the focus group discussions was 2 years (3 to 44 months). Due to the small group of possible participants, no other characteristics were collected in order to ensure confidentiality. |
| **Data collection** | | |
| 17. Interview guide | Were questions, prompts, guides provided by the authors? Was it pilot tested? | Yes |
| 18. Repeat interviews | Were repeat interviews carried out? If yes, how many? | No |
| 19. Audio/visual recording | Did the research use audio or visual recording to collect the data? | Yes |
| 20. Field notes | Were field notes made during and/or after the interview or focus group? | Yes |
| 21. Duration | What was the duration of the interviews or focus group? | focus group discussions lasted 34 minutes on average (26.5 to 45 minutes) |
| 22. Data saturation | Was data saturation discussed? | yes |
| 23. Transcripts returned | Were transcripts returned to participants for comment and/or correction? | No, transcripts were not returned due to confidentiality concerns. Final results were returned to participants for approval. |
| **Domain 3: analysis and findings** | | |
| **Data analysis** | | |
| 24. Number of data coders | How many data coders coded the data? | n=5 |
| 25. Description of the coding tree | Did authors provide a description of the coding tree? | The coding tree was discussed between coders=authors |
| 26. Derivation of themes | Were themes identified in advance or derived from the data? | Derived from the data |
| 27. Software | What software, if applicable, was used to manage the data? | n/a |
| 28. Participant checking | Did participants provide feedback on the findings? | yes |
| **Reporting** | | |
| 29. Quotations presented | Were participant quotations presented to illustrate the themes / findings? Was each quotation identified? e.g. participant number? | Yes |
| 30. Data and findings consistent | Was there consistency between the data presented and the findings? | Yes |
| 31. Clarity of major themes | Were major themes clearly presented in the findings? | Yes |
| 32. Clarity of minor themes | Is there a description of diverse cases or discussion of minor themes? | Yes |
